# Data Resource Profile Update: CPRD GOLD

**DOI:** 10.1093/ije/dyaf077

**Published:** 2025-06-11

**Authors:** Maria T Sanchez-Santos, Eleanor L Axson, Daniel Dedman, Antonella Delmestri

**Affiliations:** Centre for Statistics in Medicine, Nuffield Department of Orthopaedics, Rheumatology and Musculoskeletal Sciences, University of Oxford, Oxford, United Kingdom; Clinical Practice Research Datalink, Medicines and Healthcare products Regulatory Agency, London, United Kingdom; Clinical Practice Research Datalink, Medicines and Healthcare products Regulatory Agency, London, United Kingdom; Centre for Statistics in Medicine, Nuffield Department of Orthopaedics, Rheumatology and Musculoskeletal Sciences, University of Oxford, Oxford, United Kingdom

**Keywords:** electronic health records, primary care data, real-world data, real-world evidence

Key Features

- Clinical Practice Research Datalink (CPRD) GOLD is a large and well-regarded UK primary care data source that was established in 1987 and is used in national and international research to generate real-world evidence. Since the last-published profile, CPRD GOLD has undergone significant changes that need to be documented.
- In the last 10 years, the CPRD GOLD regional distribution of currently contributing general practitioner (GP) practices has significantly shifted, resulting in many new practices joining from Scotland, Wales, and Northern Ireland, and fewer participating from England. These changes have affected the CPRD GOLD population size, regional coverage, and eligibility for data linkages.
- CPRD GOLD January 2024 contains >21.3 million historical and current patients (12.9 in England, 3.1 in Wales, 4.7 in Scotland, 0.7 in Northern Ireland). Of these, nearly 3 million are currently registered in a GP practice and represent ∼4.3% of the estimated current UK population (0.1% in England, 32.3% in Wales, 28.6% in Scotland, 16.2% in Northern Ireland).
- Patients currently registered in CPRD GOLD January 2024 are broadly representative of the UK population with respect to age and sex.
- Future studies using CPRD GOLD should consider this updated profile to avoid potential pitfalls in study design, data interpretation, and research conclusions.

## Data resource basics

### Routinely collected data in the UK

The National Health Service (NHS) has provided freely available healthcare for the UK population since it was launched in 1948 [1]. The digital collection of its primary care data started in the late 1980s with the purpose of managing general practitioner (GP) practices and patients, and over the years has generated extremely large data sources that are used for research purposes [2]. As, in the UK, GPs play the role of NHS gatekeepers, they are also responsible for patient hospital referrals and receive outcome documentation from secondary care, which allows this information to be coded into primary care records, further enhancing the capture of patient health events. When linkages to secondary care data, mortality, disease-based or treatment-based registries, and socioeconomic status (SES) information are available, the benefits of routinely collected data for evidence-based research may expand even further, exceeding the value of using individual datasets separately [3].

### Clinical Practice Research Datalink

Clinical Practice Research Datalink (CPRD) is a not-for-profit, cost-recovery UK government research service delivered by the Medicines and Healthcare products Regulatory Agency (MHRA) with support from the National Institute for Health and Care Research (NIHR), as part of the Department of Health and Social Care [4]. CPRD collects data from three primary care electronic patient record (EPR) systems: Vision^®^, EMIS Web^®^, and TPP^®^. Vision^®^ and EMIS Web^®^ contribute to the observational databases CPRD GOLD, which includes practices across the UK, and CPRD Aurum, which hosts practices from England, respectively. Vision^®^, EMIS Web^®^, and TPP^®^ underlie the CPRD Interventional Research services.

The CPRD engagement team enrols GP practices by conducting outreach and recruitment activity across the UK. Practices using any of the supported software systems can join CPRD on a voluntary basis and can leave at any time, e.g. if they disenroll, close down, merge with another practice, or transfer to another software system. In the latter case, practices are encouraged to continue contributing to CPRD via the new system.

### CPRD GOLD

CPRD GOLD, previously known as the General Practice Research Database and Value Added Medical Products Research Databank, is a large primary care data source that was established in 1987. Its history has been widely documented [5–8] and Vision^®^, originally produced by In Practice Systems, is now part of Cegedim Healthcare Solutions. CPRD GOLD has been recognized as one of the first and largest collections of anonymized electronic health records in the UK and worldwide [9]. CPRD GOLD provides longitudinal information about patients’ demographics, symptoms, conditions, referrals, vaccinations, prescriptions, measurements, and laboratory test results collected by participating GP practices, as part of their NHS care. While, in the raw data, clinical and prescribing information is coded by using Vision^®^ bespoke coding systems (i.e. medcode and prodcode, respectively), CPRD provides dictionaries for their translation into standard coding systems (e.g. READ 3 [10] and dm+d [11], respectively). In 2015, the first data resource profile of CPRD GOLD was published [12] and its reference was added to the official CPRD GOLD documents on the CPRD website.

### Why a new CPRD GOLD profile?

In the last decade, there have been significant changes in CPRD GOLD due to the evolving market of primary care EPR systems in the UK. The Vision^®^ software system, which underlies CPRD GOLD, went from controlling 10%–20% of the UK EPR market share in 2018 and 2019 to holding only 5%–10% between 2020 and 2022. Moreover, while, in 2022, Vision^®^ held only 0–5% of the market share in England, it controlled 50%–60% in Wales, 40%–50% in Scotland, and 30%–40% in Northern Ireland [13]. This has resulted in many new GP practices joining CPRD GOLD from Scotland, Wales, and Northern Ireland, and fewer contributing from England. On the other hand, the use of EMIS Web^®^ software has grown in England, with many practices now contributing to the CPRD Aurum database [14] instead of CPRD GOLD. These variations affect the CPRD GOLD population size, regional coverage, and eligibility for data linkages, as currently CPRD can only provide linked data at the patient level for England. This paper addresses these changes and provides information about the current characteristics of CPRD GOLD. This renewed profile will help researchers to better understand the present dataset, and therefore to tailor research questions and study designs to a greater degree, avoiding possible analyses pitfalls, results misinterpretations, or inaccurate conclusions.

## Data collected

### Main change in CPRD GOLD: regional distribution

**Figure 1.**
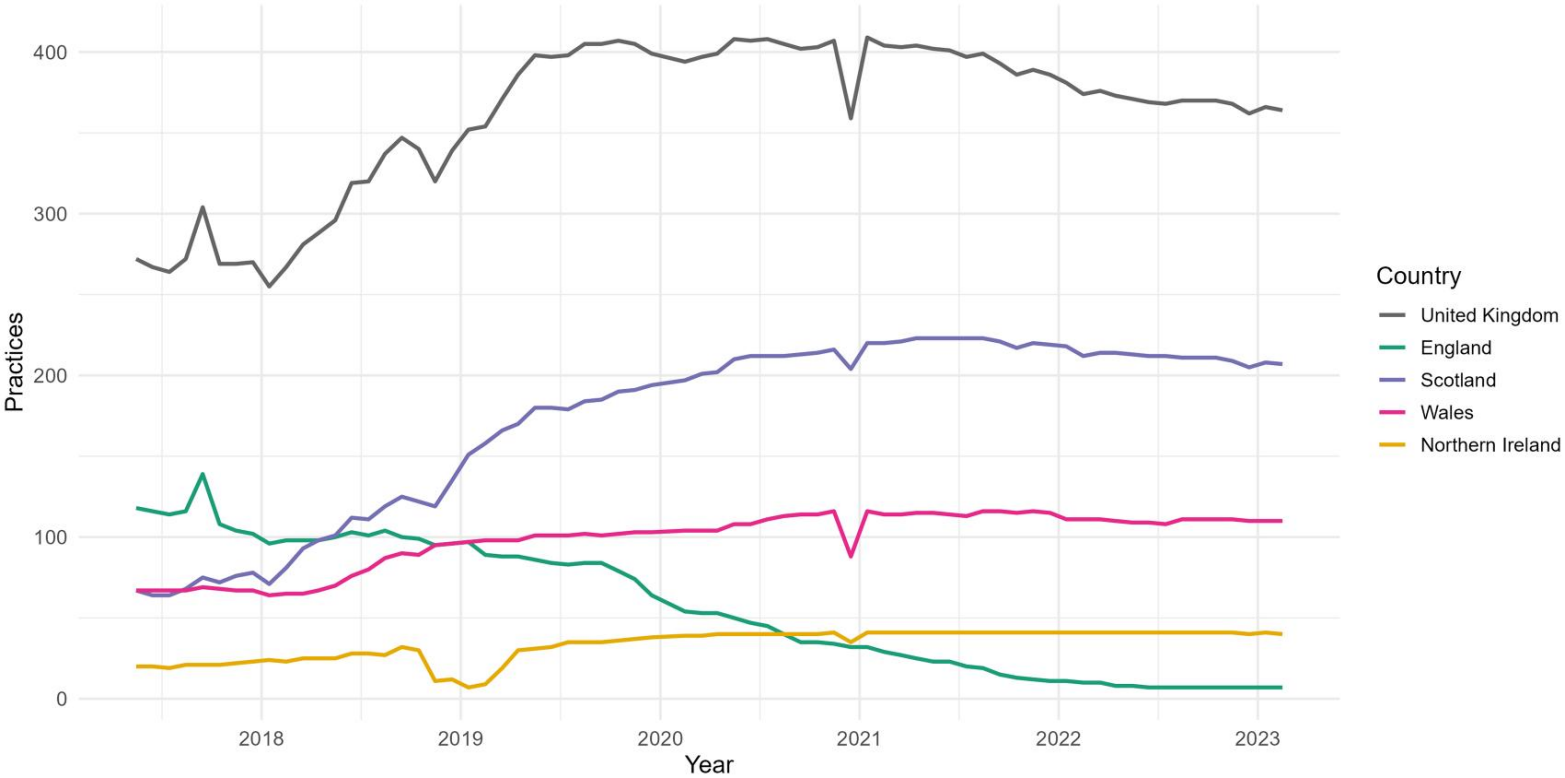
Distribution of practices contributing to CPRD GOLD by country.

The CPRD GOLD January 2024 release [15] (hereinafter CPRD GOLD Jan-2024) has 364 currently contributing GP practices (7 in England, 110 in Wales, 207 in Scotland, and 40 in Northern Ireland). Over the last decade, the practice distribution in CPRD GOLD has changed considerably for each UK constituent country: England, Wales, Scotland, and Northern Ireland (Figure 1). Regional distribution data of currently contributing practices have been provided by CPRD since April 2018 (Supplementary data).

The number of currently contributing GP practices in CPRD GOLD from England has decreased sharply over time, with a total proportional drop of 94.0% (from 118 GP practices in April 2018 to 7 in January 2024). Conversely, the numbers of GP practices from Wales, Scotland, and Northern Ireland have risen, with Scotland having the highest numbers of currently contributing practices. From April 2018 to January 2024, the UK constituent country distribution expressed as a percentage of all practices contributing to CPRD GOLD has changed from 43.4% to 1.9% in England, from 7.4% to 11.0% in Northern Ireland, from 24.6% to 30.2% in Wales, and from 24.6% to 56.9% in Scotland.

### Who is in CPRD GOLD?

CPRD GOLD Jan-2024 contains 984 historical and current GP practices from the four UK constituent countries [median of 19.1 (13.4–21.9) years of GP contribution], with coverage of >21.3 million patients. Of these, nearly 3 million are alive and currently registered in a contributing GP practice. Patients with indeterminate sex (*N* = 1137) or with year of birth prior to 1875 (*N* = 3) are excluded from the analyses. Key demographic information on current and total patients is presented in Table 1.

**Table 1.** Demographic characteristics of all acceptable (historical and current) registered patients in CPRD GOLD Jan-2024.

Characteristic	All patients (historical and current)	**Current** ^a^
Number of patients	21 377 426	2 956 992
Age in 2024 (years), [*n* (%)]		
<18	–	551 412 (18.6)
18–64	–	1 828 606 (61.8)
65+	–	576 974 (19.5)
Sex [*n* (%)]		
Female	11 077 769 (51.8)	1 492 916 (50.5)
Male	10 299 657 (48.2)	1 464 076 (49.5)
Region [*n* (%)]		
North East	205 911 (1.0)	0 (0.0)
North West	1 702 677 (8.0)	0 (0.0)
Yorkshire & The Humber	482 384 (2.3)	5611 (0.2)
East Midlands	500 564 (2.3)	0 (0.0)
West Midlands	1 377 955 (6.5)	17 016 (0.6)
East of England	1 321 576 (6.2)	0 (0.0)
London	2 469 112 (11.6)	15 925 (0.5)
South East	3 528 111 (16.5)	18 036 (0.6)
South West	1 335 499 (6.3)	0 (0.0)
Wales	3 082 628 (14.4)	1 020 735 (34.5)
Scotland	4 675 405 (21.9)	1 569 310 (53.1)
Northern Ireland	695 604 (3.3)	310 359 (10.5)

a Current patients are alive and registered in a GP practice contributing to CPRD GOLD Jan-2024.

These patients have data quality considered acceptable for clinical research and are registered in practices flagged as up-to-standard (UTS) for use in research because of their continuous high-quality data. The detailed definition of UTS practices is provided in the Supplementary data.

Patients in CPRD GOLD are labelled as ‘acceptable’ for use in research by a process that identifies and excludes patients with non-continuous follow-up or patients with poor data recording that raises suspicion as to the validity of that patient’s record. Patient data are checked, for the following issues:

- an empty or invalid first registration date;
- an empty or invalid current registration date;
- absence of a record for a year of birth;
- first registration date prior to their birth year;
- current registration date prior to their birth year;
- transferred-out reason with no transferred-out date;
- transferred-out date with no transferred-out reason;
- transferred-out date prior to their first registration date;
- transferred-out date prior to their current registration date;
- current registration date prior to their first registration date;
- gender other than female/male/indeterminate;
- age of >115 years at end of follow-up;
- recorded healthcare episodes in years prior to birth year;
- all recorded healthcare episodes have empty or invalid event dates;
- registration status of temporary patients.

If any of these conditions are true, then the patient is labelled as unacceptable and is not recommended for use in research.

CPRD GOLD Jan-2024 historical and current patients have a median follow-up of 5.9 years [interquartile range (IQR): 2.0–13.5] excluding any time before the registering practice was last flagged as UTS and 7.2 years (IQR: 2.3–18.5) without considering the UTS date. While checking the data-quality standards of GP practices at each data release supports robust clinical research, in CPRD GOLD, newly calculated UTS dates overwrite those previously recorded. This makes the identification of multiple UTS and not-UTS observation periods during a practice lifetime impossible. When using the UTS date in calculating the start of the patient observation period, 18.5% of CPRD GOLD historical and current patients are automatically excluded, as they have no observation time, while, without considering the UTS date, only 2.0% are excluded (Supplementary Tables S1 and S2). Therefore, considering or ignoring UTS dates might have an impact on studies investigating rare diseases, disease incidence/prevalence, or the trajectories of chronic conditions.

Supplementary Tables S1 and S2 give an overview of the demographic characterization of CPRD GOLD Jan-2024 historical and current patients, overall and stratified by UK constituent country, considering and ignoring UTS dates, respectively. Not considering UTS dates, CPRD GOLD Jan-2024 comprises 51.8% female patients. Regarding ethnicity (using linked data from the CPRD GOLD Ethnicity Record [16, 17]), 53.5% of patients have their ethnicity recorded. Among those, 87.8% of patients are classified as ‘White’, 6.3% as ‘Asian’, 3.2% as ‘Black’, with the remaining 2.7% from a ‘Mixed’ and ‘Other’ ethnic minority background. A similar distribution is found when UTS dates are considered.

### Representativeness—currently registered patients

#### Demographic characteristics

**Figure 2.**
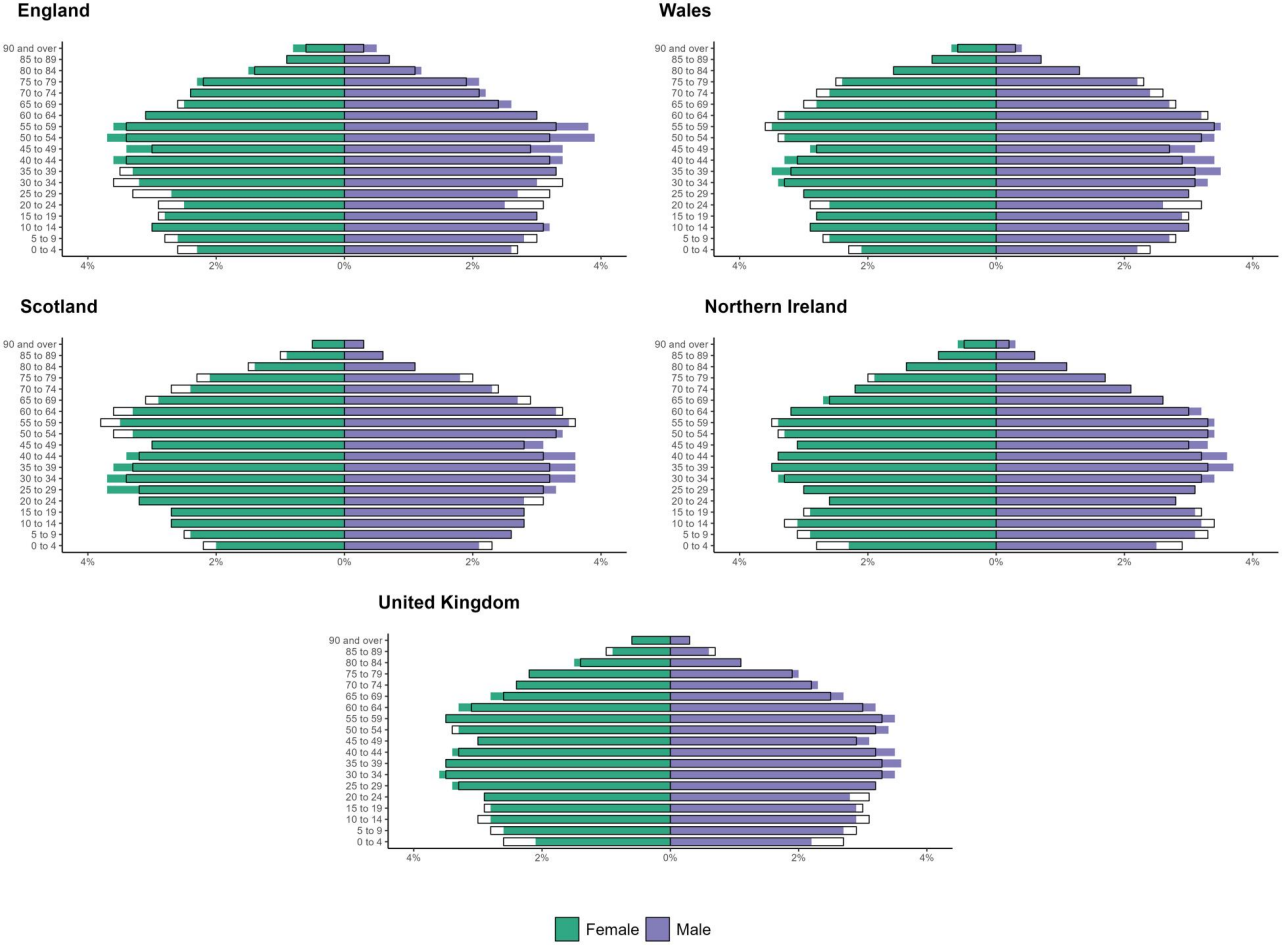
Distribution of patients by sex and 5-year age group in the CPRD GOLD Jan-2024 dataset (solid bars) in comparison with the UK mid-2023 population estimates from the ONS (outlined bars). Solid bars appearing longer or shorter than the outlined bars indicate that the demographic group in the CPRD dataset is over-represented or under-represented relative to the national population. The mid-2023 UK population estimates are rolled forward from the 2021 censuses for England, Wales, and Northern Ireland, and from the 2022 Census for Scotland; this means that population estimates from the censuses are used as the starting points for estimating the current population.

In mid-2023, the estimated UK population was ∼68.3 million (57.7 in England, 3.2 in Wales, 5.5 in Scotland, and 1.9 in Northern Ireland) [18]. CPRD GOLD Jan-2024 has 364 actively contributing practices, with ∼3 million currently registered patients (i.e. alive, registered at actively contributing GP practices, and not transferred out of the practice). These patients represent ∼4.3% of the UK population and cover 0.1% of the estimated population in England, 32.3% in Wales, 28.6% in Scotland, and 16.2% in Northern Ireland (Figure 2 and Supplementary Table S3). CPRD GOLD Jan-2024 currently registered patients have a median follow-up of 14.4 (IQR: 5.3–27.6) years without using the UTS date and 13.3 (IQR: 4.8–20.3) years when considering it.

Patients’ sex and 5-year age distributions are generally representative of the general population in the UK despite some differences. For example, data from CPRD GOLD Jan-2024 show a consistent greater representation of males in the middle age groups (30–59 years) and a lower proportion of both males and females in some of the youngest age groups (0–19 years) compared with Office for National Statistics (ONS) data (Figure 2).

When CPRD GOLD Jan-2024 is linked to CPRD GOLD Ethnicity Record [16, 17], 49.1% of all currently registered patients have ethnicity records. The proportion of patients with a recorded ethnicity is lower in Wales (38.6%), Northern Ireland (38.7%), and Scotland (56.6%) compared with England (89.1%) (Supplementary Table S4).

The ethnicity distribution of CPRD GOLD Jan-2024 is compared with ethnicity distribution from the 2021 Census in England, Wales, and Northern Ireland, and the 2022 Census in Scotland [19]. With the limitation of 50.9% missing ethnicity data (10.9% in England, 61.4% in Wales, 43.4% in Scotland, 61.3% in Northern Ireland), CPRD GOLD Jan-2024 linked to Ethnicity Record has a higher proportion of ‘White’ current patients compared with the UK population (90.7% vs 83.1%). This might be due to the populations of Wales, Scotland, and Northern Ireland being less ethnically diverse than the population of England.

#### Practice area-level socioeconomic characteristics

GP practice area-level SES [20] is measured by using the Index of Multiple Deprivation (IMD) and the Rural and Urban Classification (RUC).

In this paper, IMD and RUC measures at the practice level are used as a proxy for registered patients’ SES due to the availability of linkages for small areas at the patient level being limited to England. The distribution of both measures is shown in Table 2.

**Table 2.** IMD and RUC of currently contributing practices in CPRD GOLD Jan-2024 overall and stratified by UK constituent country.

	CPRD GOLD Jan-2024 [*n* (%)]			
Characteristic/coverage	UK	Wales	Scotland	England (E) and Northern Ireland (NI)
Number of practices	364	110	207	47
IMD practice level [*n* (%)]	2017–20	2019	2020	2019 (E) and 2017 (NI)
1—Least deprived	52 (14.3%)	10 (9.1%)	36 (17.4%)	6 (12.8%)
2	70 (19.2%)	19 (17.3%)	42 (20.3%)	9 (19.1%)
3	87 (23.9%)	26 (23.6%)	53 (25.6%)	8 (17.0%)
4	86 (23.6%)	27 (24.5%)	49 (23.7%)	10 (21.3%)
5—Most deprived	69 (19.0%)	28 (25.5%)	27 (13.0%)	14 (29.8%)
RUC practice level [*n* (%)]	2011–16	2011	2016	2011 (E) and 2015 (NI)
Urban	283 (77.7%)	88 (80.0%)	155 (74.9%)	40 (85.1%)
Rural	81 (22.3%)	22 (20.0%)	52 (25.1%)	7 (14.9%)

Due to the small number of practices from England (*N* = 7) and Northern Ireland (*N* = 40), it is recommended not to stratify by IMD or RUC individually for these countries, and therefore they have been combined in Table 2.

The representativeness of CPRD GOLD-linked IMD and RUC at the practice level in January 2024 is compared with the general population measures for each UK constituent country. Further details on how and where IMD and RUC measures of national data were obtained are available elsewhere [21] and in the Supplementary data.

The IMD in each UK constituent country population is assessed based on the distribution of national IMD quintiles, ranging from 1 (Least deprived) to 5 (Most deprived) areas. Quintiles ensure that each category includes 20% of the national population, making the distribution across the five groups even.

Overall, in the UK, there is an under-representation of GP practices in the least-deprived areas compared with the general population (IMD quintile 1: 14.3% in CPRD GOLD vs. 20% expected in the general population). Of the 364 currently contributing GP practices, 69 (19.0%) are classified as most deprived (IMD quintile 5) and 52 (14.3%) as least deprived (IMD quintile 1).

The distribution of RUC in the national population for each UK constituent country is shown in Supplementary Table S5. Table 2 shows a higher percentage of urban CPRD GOLD Jan-2024 practices in Wales compared with the total percentage of urban practices in this country (CPRD GOLD-RUC compared with national figure: 80.0% vs. 68.3%). CPRD GOLD Jan-2024 practices in Scotland are more rural compared with the total percentage of the Scottish population (25.1% vs. 17.2%).

### Data linkages

Patients from practices in England participating in the CPRD GOLD linkage scheme who did not opt out can be linked to other data sources to improve the evaluation and monitoring of patients’ health and care services. No data linkages are provided by CPRD for patients from Scotland, Wales, and Northern Ireland. For England, the actual linkage is undertaken by NHS England as a trusted third party so that no patient personal information is held by CPRD or by researchers. At present, each data source is linked by using a patient-level eight-step deterministic algorithm designed by NHS England and comes with a linkage coverage period [3, 22, 23].

These linkable data sources for England include, but are not limited to, secondary care data (e.g. Hospital Episode Statistics Admitted Patient Care, Outpatient, and Accident & Emergency), disease-based or treatment-based registries (e.g. Cancer diagnoses and Cancer treatments, COVID-19 virology tests, and hospitalizations), ONS death registration data, and socioeconomic datasets (e.g. IMD, RUC, Townsend Deprivation Index). More information about linkages available for CPRD GOLD can be found at https://www.cprd.com/cprd-linked-data.

Historical patients from England present in CPRD GOLD can be linked to the available datasets, but the small number of currently contributing English practices (*N* = 7) limits the usefulness of linked data for most studies.

### Algorithm-derived data

CPRD has developed several derived datasets based on primary care data and, if relevant/available, linked secondary care data. These datasets use algorithms to bring together and consolidate related data from throughout the patient care records [24]. Currently, three algorithm-derived datasets are available to support research using CPRD GOLD. The CPRD GOLD Pregnancy Register contains a list of all pregnancy episodes recorded in CPRD GOLD [25]. The CPRD GOLD Mother-Baby Link links likely mother–baby pairs within CPRD GOLD [26, 27]. The CPRD GOLD Ethnicity Record reports a single derived ethnicity category for each patient in CPRD GOLD [17].

## Data resource use

CPRD GOLD data have been used for decades in national and international medical research to generate real-world evidence.

The CPRD website reports >3500 peer-reviewed research publications spanning over 35 years based on CPRD GOLD and/or CPRD Aurum (https://www.cprd.com/bibliography). Up to December 2023, publications based on CPRD GOLD (alone or in combination with CPRD Aurum) represent 96.4% of all of the known CPRD bibliography. Research using CPRD GOLD data has informed pharmacovigilance, policymaking, and clinical practice, covering a wide range of clinical and therapeutic areas.

Some relevant and recent publications using CPRD GOLD include studies showing similar risk of subsequent cardiovascular events in patients with incident haemorrhagic versus ischaemic stroke [28], an increased risk of *Helicobacter pylori* infection associated with Alzheimer’s disease [29], new ethnicity-specific body mass index thresholds for obesity to prevent type 2 diabetes [30], the risk of adverse events of special interest associated with COVID-19 vaccines [31], and the accuracy of the CPRD GOLD death date for patients in England compared with the ONS mortality register [32].

## Strengths and weaknesses

CPRD GOLD has many strengths: it is a large and rich UK primary care data source whose longitudinal data collection was started in the late 1980s and is presently ongoing. The January 2024 release includes >21.3 million historical and current patients, and the latter have an average of 14.4 years of longitudinal data. CPRD GOLD had served as a data source for nearly 3500 peer-reviewed papers up to the end of 2023. It is the only data source managed by a not-for-profit service that is representative of all four UK constituent countries with respect to age and sex. In the last decade, CPRD GOLD has seen a significant increase in GP practices participating from Scotland, Wales, and Northern Ireland.

The main weakness of CPRD GOLD is the diminished presence of actively contributing English practices following variations in the UK market share of GP software systems. These changes have left only seven contributing practices using Vision^®^ software in England, with just over 46 500 currently registered patients in CPRD GOLD Jan-2024. This transformation has serious implications for patient linkage eligibility, which, for CPRD, is limited to England. While the number of historical patients eligible for linkages is considerable, the very small number of currently registered patients in England limits the utility of linkages for most studies using CPRD GOLD alone, with consequences for the range of research questions that can be answered. However, it is possible for studies to use both CPRD GOLD and CPRD Aurum, which mostly comprises English practices and therefore benefits from a large proportion of linkable data [14].

Another weakness of CPRD GOLD is the cut-off management of the practice UTS date, which does not allow the identification of multiple UTS and not-UTS observation periods during a practice lifetime. Considering UTS dates may lead to the dismissal of years of practice data when probably only a fraction of them did not meet the required quality criteria.

A limitation of this study comes from the area-level IMD and RUC measures, which, for CPRD GOLD, are only available at the practice level across the four UK constituent countries. These measures are used as proxies for patient-level measures, which are only available for patients in England. A previous study using CPRD GOLD and Aurum found that the overall agreement and correlation between practice-level and patient-level RUC were high [21].

Finally, only 49.1% of patients in CPRD GOLD Jan-2024 have linked CPRD GOLD Ethnicity Record data available, which limits the reported ethnicity distributions to fewer than half of the patients.

## Data resource access

Currently, to access CPRD data, researchers must first gain CPRD Client approval: information can be found at http://www.cprd.com/Data-access. To approved clients, CPRD offers different licence options: a single-study dataset licence or a 1-year multi-study licence. After a CPRD licence is issued, for each study, researchers need to submit a Research Application, known as a protocol, to be reviewed via CPRD’s Research Data Governance (RDG) process (http://www.cprd.com/research-applications). After protocol approval, researchers can start working with the data. More details about data access can be found at https://www.cprd.com/how-access-cprd-data.

The latest CPRD GOLD data specification, its glossary-term dictionary, and the data source profile can be found at https://www.cprd.com/primary-care-data-public-health-research.

CPRD GOLD releases are provided monthly and, for each of them, release notes, metrics, and a DOI are available at https://www.cprd.com/digital-object-identifiers-dois-datasets.

Enquiries can be submitted to CPRD via enquiries@cprd.com.

## Ethics approval

CPRD has ethics approval from the UK Health Research Authority to support research using anonymized patient data and must complete an annual NHS Data Security and Protection Toolkit assessment to demonstrate that it meets the required standard for holding data securely. Requests by researchers to access the data are reviewed via the CPRD’s RDG process to ensure that the proposed research is beneficial to patients and public health. More information is available on the CPRD website: https://www.cprd.com/safeguarding-patient-data. The current research was approved by CPRD’s RDG process (protocol number 24_003753). This study is based in part on CPRD data obtained under licence from the MHRA. These data are provided by patients and collected by the NHS as part of their care and support.

## Supplementary Material

dyaf077_Supplementary_Data
