# Supplementary material for: Data Resource Profile Update: CPRD GOLD
Source: Int J Epidemiol. 2025 Jun 11;54(4):dyaf077. doi: 10.1093/ije/dyaf077 (PMC12158158; doi:10.1093/ije/dyaf077)
Supplement: dyaf077_Supplementary_Data [file dyaf077_supplementary_data.zip › ije-2024-10-1530-File006.pdf]

SUPPLEMENTARY INFORMATION

Up-to-Standard (UTS) practice definition in Clinical Practice Research Datalink (CPRD) GOLD

The overall quality of data in practices in CPRD GOLD is mediated by use of an 'up-to-standard' (UTS) date, which is deemed as the date at which data in the practice is considered to have continuous high-quality data fit for use in research. This is mediated by an analysis on the total data in the practice, which is refreshed every time a new collection for a practice is processed into the database. It is based on two central concepts: assurance of continuity in data recording (gap analysis), and avoidance of use of data for which transferred out and dead patients have been removed (death recording).

Gap Analysis

To detect whether there are any meaningful gaps in the data it is necessary to look in more detail at single day gaps as well as longer gaps. A single day alone may reflect a situation where nothing was recorded that day at the practice, i.e. the practice was not open, such as on a bank holiday. A longer gap may reflect a situation where the practice did not offer a service, and patients may have been treated elsewhere. If a meaningful gap is found, the earliest date after which there is no significant gap is identified.

Death Recording

It is expected that a standard number of deaths will be recorded at a practice over time. Assessment of gaps in death recording is performed taking the size of the practice into account. A safety margin is built into account for both geographical and seasonal variation in death rates. If a meaningful gap is found, the earliest date after which there is no significant gap is identified.

The UTS date is set to the latest of these dates for each practice. The CPRD recommend that analyses are performed on data following the practice UTS date.

Table S1. Demographic characteristics of all acceptable (historical and current) registered patients considering up-to-standard (UTS) date in Clinical Practice Research Datalink (CPRD) GOLD Jan-2024 overall and stratified by United Kingdom (UK) constituent country.

	**UK**	**England**	**Wales**	**Scotland**	**Northern Ireland**
**Characteristic**	**N=17 640 072**	**N=10 686 856**	**N=2 535 122**	**N=3 816 364**	**N=601 730**
Follow-up, median (IQR)	5.85 (1.98 - 13.45)	5.46 (1.92 - 12.32)	6.84 (2.12 - 15.45)	5.86 (1.89 - 14.92)	10.86 (3.50 - 19.91)
Records excluded due to start ≥ end observation, n (%)	3 965 375 (18.5%)	2 426 857 (18.8%)	556 060 (18.0%)	886 706 (19.0%)	95 752 (13.8%)
Sex, n (%)					
Female	9 075 613 (51.4%)	5 505 859 (51.5%)	1 299 448 (51.3%)	1 960 663 (51.4%)	309 643 (51.5%)
Male	8 564 459 (48.6%)	5 180 997 (48.5%)	1 235 674 (48.7%)	1 855 701 (48.6%)	292 087 (48.5%)
**Individuals with ethnicity data available (n, % of the total)**	**10 429 549 (59.1%)**	**7 597 024 (71.1%)**	**801 568 (31.6%)**	**1 841 985 (48.3%)**	**188 972 (31.4%)**
Ethnicity*, n (%)					
White	9 211 231 (88.3%)	6 645 307 (87.5%)	726 620 (90.6%)	1 663 962 (90.3%)	175 342 (92.8%)
Black	316 939 (3.0%)	266 798 (3.5%)	14 494 (1.8%)	33 049 (1.8%)	2 598 (1.4%)
Asian	632 524 (6.1%)	491 984 (6.5%)	41 372 (5.2%)	91 305 (5.0%)	7 863 (4.2%)
Mixed/Multiple	160 191 (1.5%)	131 034 (1.7%)	9 763 (1.2%)	18 278 (1.0%)	1 116 (0.6%)
Other	108 664 (1.0%)	61 901 (0.8%)	9 319 (1.2%)	35 391 (1.9%)	2 053 (1.1%)

Note: Records where the start of observation is higher or equal to the end of observation were excluded for the follow-up calculation. 1 137 patients with indeterminate or unknown sex and 3 patients with year of birth < 1875 were excluded.

UTS = up-to-standard for practice data quality.

*Ethnicity comes from linked CPRD GOLD Ethnicity Record

Table S2. Demographic characteristics of all acceptable (historical and current) registered patients without considering up-to-standard (UTS) date in Clinical Practice Research Datalink (CPRD) GOLD Jan-2024 release overall and stratified by United Kingdom (UK) constituent country.

	**UK**	**England**	**Wales**	**Scotland**	**Northern Ireland**
**Characteristic**	**N=21 377 426**	**N=12 923 789**	**N=3 082 628**	**N=4 675 405**	**N=695 604**
Follow-up, median (IQR)	7.20 (2.32 - 18.49)	6.84 (2.33 - 17.00)	7.87 (2.19 - 20.15)	7.19 (2.21 - 20.66)	14.29 (4.13 - 28.00)
Records excluded due to start ≥ end observation, n (%)	427 080 (2.0%)	196 140 (1.5%)	67 595 (2.2%)	151 772 (3.2%)	11 573 (1.7%)
Sex, n (%)					
Female	11 077 769 (51.8%)	6 708 648 (51.9%)	1 589 401 (51.6%)	2 417 509 (51.7%)	362 211 (52.1%)
Male	10 299 657 (48.2%)	6 215 141 (48.1%)	1 493 227 (48.4%)	2 257 896 (48.3%)	333 393 (47.9%)
**Individuals with ethnicity data available (n, % of the total)**	**11 445 289 (53.5%)**	**8 456 791 (65.4%)**	**842 940 (27.3%)**	**1 956 466 (41.8%)**	**189 092 (27.2%)**
Ethnicity*, n (%)					
White	10 051 124 (87.8%)	7 364 308 (87.1%)	758 194 (89.9%)	1 753 180 (89.6%)	175 442 (92.8%)
Black	366 116 (3.2%)	312 631 (3.7%)	16 036 (1.9%)	34 847 (1.8%)	2 602 (1.4%)
Asian	720 332 (6.3%)	557 944 (6.6%)	47 104 (5.6%)	107 406 (5.5%)	7 878 (4.2%)
Mixed/Multiple	179 177 (1.6%)	148 174 (1.8%)	10 271 (1.2%)	19 615 (1.0%)	1 117 (0.6%)
Other	128 540 (1.1%)	73 734 (0.9%)	11 335 (1.3%)	41 418 (2.1%)	2 053 (1.1%)

Note: Records where the start of observation is higher or equal to the end of observation were excluded for the follow up calculation. 1 137 patients with indeterminate or unknown sex and 3 patients with year of birth < 1875 were excluded. UTS = up-to-standard for practice data quality.

*Ethnicity comes from linked CPRD GOLD Ethnicity Record

Table S3. Sex and age distribution of currently registered patients in Clinical Practice Research Datalink (CPRD) GOLD Jan-2024 overall and stratified by United Kingdom (UK) constituent countries compared to mid-2023 Office for National Statistics (ONS) national data.

	CPRD GOLD, n (%)					UK population estimates from mid-2023 ONS				
**Characteristic**	**UK**	**England**	**Wales**	**Scotland**	**Northern Ireland**	**UK**	**England**	**Wales**	**Scotland**	**Northern Ireland**
**No. of individuals**	**2 956 992**	**56 588**	**1 020 735**	**1 569 310**	**310 359**	**68 265 209**	**57 690 323**	**3 164 404**	**5 490 100**	**1 920 382**
Age in 2024, n (%)										
0-19	615 779 (20.8%)	12 583 (22.2%)	217 400 (21.3%)	314 155 (20.0%)	71 641 (23.1%)	15 659 683 (22.9%)	13 347 407 (23.1%)	695 404 (22.0%)	1 134 033 (20.7%)	482 839 (25.1%)
20-44	984 814 (33.3%)	17 067 (30.2%)	323 758 (31.7%)	542 671 (34.6%)	101 318 (32.6%)	22 308 306 (32.7%)	18 981 946 (32.9%)	970 002 (30.7%)	1 755 693 (32.0%)	600 665 (31.3%)
45-64	779 425 (26.4%)	15 784 (27.9%)	266 616 (26.1%)	415 149 (26.5%)	81 876 (26.4%)	17 373 054 (25.4%)	14 577 883 (25.3%)	816 762 (25.8%)	1 484 013 (27.0%)	494 396 (25.7%)
65 and older	576 974 (19.5%)	11 154 (19.7%)	212 961 (20.9%)	297 335 (18.9%)	55 524 (17.9%)	12 924 166 (18.9%)	10 783 087 (18.7%)	682 236 (21.6%)	1 116 361 (20.3%)	342 482 (17.8%)
Sex, n (%)										
Female	1 492 916 (50.5%)	28 439 (50.3%)	513 914 (50.3%)	795 758 (50.7%)	154 805 (49.9%)	34 814 781 (51.0%)	29 407 249 (51.0%)	1 611 486 (50.9%)	2 821 149 (51.4%)	974 897 (50.8%)
Male	1 464 076 (49.5%)	28 149 (49.7%)	506 821 (49.7%)	773 552 (49.3%)	155 554 (50.1%)	33 450 428 (49.0%)	28 283 074 (49.0%)	1 552 918 (49.1%)	2 668 951 (48.6%)	945 485 (49.2%)

Currently refers to patients who are alive and registered at actively contributing practices. The mid-2023 UK population estimates for age and sex are rolled forward from the 2021 censuses for England, Wales, Northern Ireland, and from the 2022 Census for Scotland; this means that population estimates from the censuses are used as the starting points for estimating the current population.

Table S4. Ethnicity distribution of currently registered patients in Clinical Practice Research Datalink (CPRD) GOLD Jan-2024 overall and stratified by United Kingdom (UK) constituent countries compared to national data from UK 2021/2022 censuses.

	**CPRD GOLD, n (%)**					**National Data from UK censuses 2021/2022, n (%)**				
**Ethnicity**	**UK**	**England**	**Wales**	**Scotland**	**Northern Ireland**	**UK**	**England**	**Wales**	**Scotland**	**Northern Ireland**
**No. of individuals with ethnicity data available (% of the total)**	**1 453 041 (49.1%)**	**50 408 (89.1%)**	**394 093 (38.6%)**	**888 294 (56.6%)**	**120 246 (38.7%)**	**66 937 100**	**56 489 800**	**3 107 500**	**5 436 600**	**1 903 200**
Ethnicity, n (%)	[a]					[b]				
White	1 317 693 (90.7%)	44 130 (87.5%)	361 597 (91.8%)	798 659 (89.9%)	113 307 (94.2%)	55 592 835 (83.1%)	45 783 401 (81.0%)	2 915 848 (93.8%)	5 051 873 (92.9%)	1 841 713 (96.8%)
Black	23 676 (1.6%)	523 (1.0%)	6 490 (1.6%)	15 183 (1.7%)	1 480 (1.2%)	2 485 724 (3.7%)	2 381 724 (4.2%)	27 554 (0.9%)	65 414 (1.2%)	11 032 (0.6%)
Asian	74 754 (5.1%)	4 547 (9.0%)	17 817 (4.5%)	48 612 (5.5%)	3 778 (3.1%)	5 758 109 (8.6%)	5 426 392 (9.6%)	89 028 (2.9%)	212 022 (3.9%)	30 667 (1.6%)
Mixed/multiple	15 156 (1.0%)	991 (2.0%)	4 306 (1.1%)	9 234 (1.0%)	625 (0.5%)	1 793 257 (2.7%)	1 669 378 (3.0%)	48 598 (1.6%)	60 899 (1.1%)	14 382 (0.8%)
Other	21 762 (1.5%)	217 (0.4%)	3 883 (1.0%)	16 606 (1.9%)	1 056 (0.9%)	1 308 819 (2.0%)	1 229 153 (2.2%)	26 466 (0.9%)	49 632 (0.9%)	3 568 (0.2%)

Currently refers to patients who are alive and registered at actively contributing practices. The mid-2023 UK population estimates for age and sex are rolled forward from the 2021 censuses for England, Wales, Northern Ireland, and from the 2022 Census for Scotland; this means that population estimates from the censuses are used as the starting points for estimating the current population.

[a]Ethnicity comes from linked CPRD GOLD Ethnicity Records

[b]The general population of the UK in Census 2021 in each higher-level ethnic category obtained from the combined figures from 2021-2022 Census in England and Wales, Northern Ireland, and Scotland

What has been measured?

CPRD GOLD regional distribution change

CPRD GOLD regions are listed in the practice Region (PRG) lookup file and associated to each practice in the Practice file. The CPRD GOLD regional distribution of currently contributing practices was captured using monthly CPRD GOLD release notes from April 2018 to January 2024 (see Supplementary data - File "*cprd_gold_regional_distribution_201804_202401.csv*"). Beforehand, no regional distribution of currently contributing practices was reported by CPRD.

CPRD GOLD demographic characteristics

Age (represented by the year of birth) and sex were based on data recorded in the CPRD GOLD Patient file and calculated for historical and current patients.

Ethnicity was reported using the CPRD GOLD Ethnicity Record (i.e. Asian, Black, Mixed/Multiple, White, Other, and Unknown) for historical and current patients (CPRD GOLD Ethnicity Record Documentation https://cprd.com/sites/default/files/2023-05/CPRD_GOLD_EthnicityRecord_Documentation_v1.0.pdf).

General practitioner (GP) practice area level deprivation was measured by using the most recent Index of Multiple Deprivation (IMD), and the rural-urban classification (RUC). These measurements are based on GP practice postcode, available for all practices in CPRD GOLD. IMD was reported in quantiles (1=Least deprived), and RUC as a binary variable (1=Urban, 2=Rural) (https://cprd.com/sites/default/files/2022-05/Documentation_SmallAreaData_Practice_set22_v3.4_1.pdf)

National data sources by United Kingdom (UK) constituent countries

To evaluate the representativeness of CPRD GOLD, we compared the current CPRD GOLD Jan-2024 population to the UK general population, overall and by UK constituent country.

Further details on the data sources used by UK constituent country are available below and in Table S6.

Demographic characteristics

Age and sex data for the UK general population were obtained from the 2023 mid-year population estimates. Ethnicity was collected from the 2021 censuses for England, Wales, and Northern Ireland, and from the 2022 Census for Scotland:

- The Office for National Statistics (ONS) 2021 census in England and Wales: https://www.ons.gov.uk/census.
- Northern Ireland Statistics and Research Agency (NISRA) for Census 2021 in Northern Ireland: https://www.nisra.gov.uk/statistics/census/2021-census.
- National Records of Scotland (NRS) for Census 2022 in Scotland: https://www.scotlandscensus.gov.uk/about/2022-census/.

Area-level socioeconomic measures

All small areas in each UK constituent country are ranked according to their level of deprivation.

The IMD is an overall relative measure of deprivation calculated by combining the weighted indices of deprivation from several domains (e.g. income, employment, education, health, crime, access to services, and living environment). The number of domains and indicators are slightly different across each constituent country in the UK.

IMD is measured and reported at lower-layer super output areas (LSOAs) for England and Wales, data zones (DZs) for Scotland, and super output areas (SOAs) for Northern Ireland (NI). Below is the list of references with the IMD information for each UK constituent country:

- Ministry of Housing, Communities & Local Government. The English indices of deprivation 2019. Available at: https://www.gov.uk/government/statistics/english-indices-of-deprivation-2019

- Statistics for Wales, Welsh Government. Welsh index of multiple deprivation (WIMD), 2019: Available at: https://www.gov.wales/welsh-index-multiple-deprivation-full-index-update-ranks-2019
- Scottish Government. Scottish index of multiple deprivation (SMID) 2020. Available at: https://www.gov.scot/collections/scottish-index-of-multiple-deprivation-2020/
- Northern Ireland Statistics & Research Agency (NISRA). Northern Ireland multiple deprivation measure 2017 (NIMDM2017). Available at: https://www.nisra.gov.uk/statistics/deprivation/northern-ireland-multiple-deprivation-measure-2017-nimdm2017

National IMD quintiles (ranked separately for England, Wales, Scotland and NI) are used in this profile study.

RUC is a measure of urbanisation, based on the size of the resident population only within a specific geographical area, and does not consider land use, policy or financial characteristics of an area. This measure is used to categorise patient residences as either primarily 'rural' or 'urban' as a binary variable. Areas are classified as rural if they are located outside of settlements with a population exceeding 10,000 residents. The RUC distribution of national figures for each UK constituent country is shown in Table S5 below.

Table S5. National Rural and Urban Classification (RUC) data

	**National RUC classification, %**			
	**England, 2011**	**Wales, 2011**	**Scotland, 2016**	**Northern Ireland, 2015**
Urban	83.0%	68.3%	82.8%	70.0%
Rural	17.0%	31.7%	17.2%	30.0%

For details on how RUC was created in each UK constituent country, see the list of references below:

- Ministry of Housing, Communities & Local Government. Office for National Statistics. 2011 Rural Urban Classification in England and Wales. Available at: https://www.gov.uk/government/statistics/2011-rural-urban-classification-lookup-tables-for-all-geographies
- Scottish Government. Scottish Government Urban Rural Classification 2016. Available at: https://www.gov.scot/publications/scottish-government-urban-rural-classification-2016/
- Northern Ireland Statistics and Research Agency (NISRA). Northern Ireland Urban-Rural Classification 2015. Available at: https://www.nisra.gov.uk/support/geography/urban-rural-classification

Table S6**.** Demographic variables available from national data sources by United Kingdom (UK) constituent country

Variables	England	Wales	Scotland	Northern Island
Age	<u>Source</u>: *Census 2021*<br>Age is derived from date of birth question and the age of the person on census date (21 March 2021)	<u>Source</u>: *Census 2021*<br>Age is derived from date of birth question and the age of the person on census date (21 March 2021)	<u>Source</u>: *Census 2022*<br>Age is derived from date of birth question and the age of the person on census day (20 March 2022)	<u>Source</u>: *Census 2021*<br>Age is derived from date of birth question and the age of the person on census date (21 March 2021)
Sex	<u>Source</u>: *Census 2021*<br>What is your sex?<br>• Female<br>• Male	<u>Source</u>: *Census 2021*<br>What is your sex?<br>• Female<br>• Male	<u>Source</u>: *Census 2022*<br>What is your sex?<br>• Female<br>• Male	<u>Source</u>: *Census 2021*<br>What is your sex?<br>• Female<br>• Male
Ethnicity	<u>Source</u>: *Census 2021*<br>What is your ethnic group?<br>• White<br>• Mixed or Multiple ethnic group<br>• Asian or Asian British<br>• Black, Black British, Caribbean or African<br>• Other ethnic group	<u>Source</u>: *Census 2021*<br>What is your ethnic group?<br>• White<br>• Mixed or Multiple ethnic group<br>• Asian, Asian Welsh or Asian British<br>• Black, Black Welsh, Black British, Caribbean or African<br>• Other ethnic group	<u>Source</u>: *Census 2022*<br>What is your ethnic group?<br>• White<br>• Mixed or Multiple ethnic group<br>• Asian, Scottish Asian, or British Asian<br>• Black (African, Scottish African, or British African + Caribbean or Black)<br>• Other ethnic group	<u>Source</u>: *Census 2021*<br>What is your ethnic group?<br>• White (White + Irish Traveller + Roma)<br>• Mixed ethnic group<br>• Asian (Indian, Chinese, Filipino, Pakistan, other Asian)<br>• Black (Black African + Black other)<br>• Any other ethnic group
Area-level measures (SES and RUC)	<u>Source</u>: *National-level IMD 2019 and RUC 2011 data* (postcode linked via LSOA)<br>IMD (1=Least deprived)<br>RUC (Rural & Urban)	<u>Source</u>: *National-level IMD 2019 and RUC 2011 data* (postcode linked via LSOA)<br>IMD (1=Least deprived)<br>RUC (Rural & Urban)	<u>Source</u>: *National-level IMD 2020 and RUC 2016 data* (postcode linked via DZ)<br>IMD (1=Least deprived)<br>RUC (Rural & Urban)	<u>Source</u>: *National-level IMD 2017 and RUC 2015 data* (postcode linked via SOA)<br>IMD (1=Least deprived)<br>RUC (Rural & Urban)

SES: Socioeconomic status; LSOA: lower layer Super Output Area; SOA: Super Output Area; DZ: datazone; IMD: Index of Multiple Deprivation; RUC: Rural and Urban classification.
